# Outcomes of Patients Who Developed Clostridioides difficile Infection During Hospitalization and Had a History of Comorbid Post-Traumatic Stress Disorder

**DOI:** 10.7759/cureus.28810

**Published:** 2022-09-05

**Authors:** Shivani J Patel, Alexander Kaye, Sarah R Meyers, Sushil Ahlawat

**Affiliations:** 1 Internal Medicine, Rutgers University New Jersey Medical School, Newark, USA; 2 Psychiatry, Rutgers Robert Wood Johnson Medical School, Piscataway, USA; 3 Gastroenterology and Hepatology, Rutgers University New Jersey Medical School, Newark, USA

**Keywords:** sepsis, inpatient mortality, posttraumatic stress disorders, c.difficile, clostridioides difficile infection

## Abstract

Introduction:* Clostridioides difficile *(*C. difficile*), is a common cause of nosocomial diarrhea. Antibiotic use is a risk factor for developing *C. difficile* infection (CDI). Clinical presentations of CDI range from mild diarrhea to fulminant colitis. A history of anxiety increases the risk of developing irritable bowel syndrome following CDI. Post-traumatic stress disorder (PTSD) is a common form of anxiety and is associated with several medical comorbidities. This study explores the impact PTSD has on the outcomes of adult patients who develop CDI while hospitalized.

Methods: Hospitalized adults who had developed CDI were selected from the 2014 National Inpatient Sample database using the International Classification of Diseases, 9th Revision, Clinical Modification (ICD-9) codes. The outcomes of CDI patients with and without comorbid PTSD were explored. The outcomes assessed in this study were inpatient mortality, sepsis, hypotension/shock, acute renal failure, acute respiratory failure, megacolon, colonic perforation, and intestinal abscess. Independent t-tests and chi-squared tests were used to compare means and proportions, respectively. A multivariate logistic regression analysis was utilized to determine whether PTSD is an independent predictor of the outcomes.

Results: Among 72,383 hospitalized adults who developed CDI in the year 2014, 465 also had a diagnosis of PTSD. PTSD was found to be an independent risk factor for inpatient mortality (adjusted odds ratio {aOR} 2.93, 95% confidence interval (CI): 1.39-6.21, p = 0.005), and sepsis (aOR 1.61, 95% CI: 1.24-2.07, p = 0.001). However, PTSD was not a risk factor for hypotension/shock (aOR 1.26, 95% CI: 0.97-1.63, p = 0.080), acute renal failure (aOR 1.02, 95% CI: 0.81-1.28, p = 0.895), or acute respiratory failure (aOR 1.15, 95% CI: 0.83-1.58, p = 0.412) in patients with CDI. Due to small sample sizes of patients who developed megacolon, colonic perforation, and intestinal abscess, further analysis of these outcomes was not performed.

Conclusion: Inpatients who develop CDI with comorbid PTSD are at increased risk for sepsis and inpatient mortality. These findings may be due to the impact of PTSD’s dysregulation of the hypothalamic-pituitary axis leading to low cortisol production, increased serum cytokine concentrations, and/or increased intestinal inflammation. Awareness of these increased risks when triaging CDI patients with PTSD and possibly increased psychiatric interventions to treat PTSD may be necessary to help reduce the risk of sepsis and inpatient mortality in this subgroup of patients.

## Introduction

*Clostridioides difficile *(*C. difficile*) is a spore-forming, anaerobic gram-positive bacillus and is a common cause of nosocomial infections [1]. *C. difficile* was initially identified in 1978. The discovery corresponded to the observation of clindamycin-induced diarrhea in 21% of patients; many of these cases could be attributed to *C. difficile *infection (CDI) [2,3]. With the rise in the use of antibiotics and the availability of new types of antibiotics, CDI prevalence had dramatically increased, and it has become one of the most widespread nosocomial infections [2]. Approximately one to three percent of healthy adults and 20% of adults with a history of antibiotic exposure are colonized with *C. difficile* [4]. *C. difficile* spores are often transmitted from colonized individuals to uncolonized patients by healthcare workers and visitors to healthcare facilities [1]. Patients on antibiotics are at risk for gut microbiome disruption and reduction of microflora diversity, increasing the risk of gastrointestinal colonization by *C. difficile* [1]. The release of toxins produced by *C. difficile* causes cytotoxic and cytopathic cell damage and activation of the inflammation cascade resulting in CDI and diarrhea [1]. In addition to antibiotic exposure, risk factors for CDI include age older than 65 years, prolonged hospitalization, and co-existing diagnosis of inflammatory bowel disease (IBD) [2].

The clinical presentation of CDI can range from mild diarrhea to fulminant colitis [2]. Additional severe complications that can arise from CDI include circulatory shock, toxic megacolon, colonic perforation, ileus, acute kidney failure, sepsis, and death [2]. The Infectious Diseases Society of America and European Society of Clinical Microbiology and Infectious Diseases recommend making the diagnosis of CDI based on an initial stool DNA test or stool antigen test, followed by a confirmatory *C. difficile* toxin A/B enzyme immunoassay [2,5]. The preferred pharmacologic agents for management include vancomycin or fidaxomicin [2,5]. Fecal microbiota transplant can be considered in the setting of recurrent CDI. Surgical management is reserved for severely ill patients with toxic megacolon [2,5]. After CDI, patients are at higher risk for new onset irritable bowel syndrome (IBS), especially if they had a pre-existing anxiety diagnosis [6].

In the United States, a common mental health diagnosis is post-traumatic stress disorder (PTSD). This disorder was initially believed to affect predominantly military personnel, however, 6.8% of the general population have been diagnosed with it [7]. This disorder is instigated by a traumatic event or stressor, later leading to symptoms of intrusive thoughts, alteration in mood and cognition, avoidance of certain environmental exposures, hypervigilance, and reactivity [8]. The pathogenesis of PTSD has been posited to be due to alterations in the hypothalamic-pituitary axis (HPA) and the sympathetic nervous system (SNS) [8,9]. Treatment of PTSD can include cognitive behavior therapy, selective serotonin reuptake inhibitors, monoamine oxidase inhibitors, and alpha-blockers [8]. Recent studies are also further elucidating the mind-gut axis and the use of selective serotonin reuptake inhibitors (SSRIs) has been associated with alterations of the gut microbiome in animal models [10]. Furthermore, patients with PTSD are also notably at higher risk of physical health comorbidities, such as heart disease [11]. Despite these new studies, little research has directly explored the impact of psychiatric disorders on patients with gastrointestinal pathology on clinical outcomes. This study explored the outcomes of adult patients with a history of PTSD who developed CDI during hospitalization. 

## Materials and methods

A retrospective cohort study was performed involving inpatient adults (defined as 18 years old or older) who were diagnosed with CDI during their hospital stay in the year 2014. The patient population for this study was derived from the National Inpatient Sample (NIS), Healthcare Cost and Utilization Project (HCUP), and the Agency for Healthcare Research and Quality, which is widely understood to be the largest all-payer inpatient database in the United States [12]. All patient outcomes and diagnoses assessed in the study were identified within the NIS database using the International Classification of Diseases, Ninth Edition Revision, Clinical Modification (ICD-9) codes. After identifying all adult inpatients during the year 2014 who had developed CDI, this group was then divided into a subgroup with comorbid PTSD and a subgroup without comorbid PTSD. Data about each of the subgroups’ demographics and hospitalizations including age, sex, race, hospitalization cost, and length of hospital stay were extracted from the NIS data and subsequently compared between the subgroup with PTSD and the subgroup without PTSD. The Charlson Comorbidity Index, a standardized predictor of 10-year mortality based on multiple comorbidities, was calculated for both subgroups and compared [13,14].

SPSS Statistics, version 28.0 (IBM Corp., Armonk, NY) was utilized for all statistical analyses performed in this study. The outcomes assessed in this study included inpatient mortality, sepsis, hypotension/shock, acute renal failure, acute respiratory failure, megacolon, colonic perforation, and intestinal abscess. These outcomes were compared between the two subgroups. Independent t-tests and chi-squared tests were used to compare means and proportions, respectively. The statistical analyses conducted were two-tailed, and a p-value of below 0.05 was considered to be statistically significant. Continuous variables were reported as means ± standard deviations (SDs) while categorical variables were reported as numbers (N) and percentages (%). Additionally, a multivariate logistic regression analysis was performed to explore whether PTSD was an independent predictor for the clinical outcomes, after detecting, adjusting, and controlling for confusion factors such as age, sex, race, and Charlson Comorbidity Index.

## Results

Over the course of the year 2014, 72,383 hospitalized adults in the United States were found to have developed CDI. Among all patients who developed CDI, 465 also had a comorbid diagnosis of PTSD. As demonstrated in Table 1, the PTSD subgroup was younger (50.5 years vs. 65.1 years, p < 0.001), and had a lower Charlson Comorbidity Index (2.56 vs. 4.50, p < 0.001). There was no statistically significant difference in sex (p = 0.344), race (p = 0.212), length of hospital stay (p = 0.504), and total hospital charges (p = 0.888) between the two subgroups. 

**Table 1:** Demographics, characteristics, length of stay, total hospital charge, and Charles Comorbidity Index among Clostridioides difficile patients with and without a history of post-traumatic stress disorder *Exact number is not included in the table due to database guidelines not allowing for the reporting of a sample size of fewer than 10 patients.

Variable	With posttraumatic stress disorder	Without posttraumatic stress disorder	p-value
N = 72,383	N = 465	N = 71,918	
Patient age, mean (SD)	50.5 (16.0)	65.1 (19.4)	<0.001
Sex, N (%)			0.344
Female	261 (56.1%)	41,929 (58.3%)	
Male	204 (43.9%)	29,989 (41.7%)	
Race, N (%)			0.212
White	341(78.0%)	50,721 (74.0%)	
Black	41 (9.4%)	8,849 (12.9%)	
Hispanic	35 (8.0%)	5,524 (8.1%)	
Asian or Pacific Islander	*	1,377 (2.0%)	
Native American	*	461 (0.7%)	
Other	12 (2.7%)	1,587 (2.3%)	
Length of stay, in days (SD)	10.1 (12.5)	10.6 (13.9)	0.504
Total hospital charges, in $ (SD)	91,503 (209,205)	92,890 (176,441)	0.888
Charlson Comorbidity Index (SD)	2.56 (2.44)	4.50 (2.74)	<0.001

In Table 2, the outcomes of CDI patients with and without a co-diagnosis of PTSD were compared. Those patients with comorbid PTSD were found to have decreased inpatient mortality (2.2% vs. 6.7%, p < 0.001), decreased sepsis (17.0% vs. 27.5%, p < 0.001), decreased hypotension/shock (15.9% vs. 21.5%, p = 0.003), and decreased acute renal failure (23.4% vs. 31.2%, p = 0.002). No statistically significant differences were present between the prevalence of acute respiratory failure (p = 0.089), megacolon (p = 0.376), colonic perforation (p = 0.962), and intestinal abscess (p = 0.516). Given the small sample sizes of megacolon, colonic perforation, and intestinal abscess, further analysis of these outcomes was not performed.

**Table 2:** Unadjusted clinical outcomes among Clostridioides difficile patients with and without a history of post-traumatic stress disorder *Exact number is not included in the table due to database guidelines not allowing for the reporting of a sample size of fewer than 10 patients.

Outcomes	With posttraumatic stress disorder	Without posttraumatic stress disorder	p-value
Inpatient mortality	10 (2.2%)	4,827 (6.7%)	<0.001
Sepsis	79 (17%)	19,774 (27.5%)	<0.001
Hypotension/shock	74 (15.9%)	15,497 (21.5%)	0.003
Acute renal failure	109 (23.4%)	22,457 (31.2%)	0.002
Acute respiratory failure	44 (9.5%)	8,654 (12.0%)	0.089
Megacolon	*	121 (0.2%)	0.376
Colonic perforation	*	320 (0.4%)	0.962
Intestinal abscess	*	293 (0.4%)	0.516

Table 3 displays the odds ratios of the clinical outcomes after they had been adjusted for sex, race, age, and Charlson Comorbidity Index. PTSD was found to be an independent risk factor for inpatient mortality (adjusted odds ratio {aOR} 2.93, 95% confidence interval (CI): 1.39-6.21, p = 0.005), and sepsis (aOR 1.61, 95% CI: 1.24-2.07, p = 0.001). However, the aORs for hypotension/shock (aOR 1.26, 95% CI: 0.97-1.63, p = 0.080), acute renal failure (aOR 1.02, 95% CI: 0.81-1.28, p = 0.895), and acute respiratory failure (aOR 1.15, 95% CI: 0.83-1.58, p = 0.412) were not found to be statistically significant. Despite Table 2 and Table 3 containing the same outcomes, the data may appear to conflict. For example, the outcomes, sepsis and inpatient mortality, in Table 2 were seen less commonly in the PTSD subgroup. On the other hand, in Table 3, sepsis and inpatient mortality occurred more commonly in patients with PTSD. These differences can be explained by confounding factors that were adjusted for in Table 3.

**Table 3:** Multivariate logistic regression analysis of clinical outcomes among Clostridioides difficile patients with and without a history of post-traumatic stress disorder *Adjusted for age, sex, race, and the Charlson Comorbidity Index.

Outcomes	Adjusted odds ratio*	95% Confidence Interval	p-value
Inpatient mortality	2.93	1.39-6.21	0.005
Sepsis	1.61	1.24-2.07	0.001
Hypotension/shock	1.26	0.97-1.63	0.080
Acute renal failure	1.02	0.81-1.28	0.895
Acute respiratory failure	1.15	0.83-1.58	0.412

## Discussion

The results of this study revealed that patients who develop CDI with a history of comorbid PTSD are at elevated risk of inpatient mortality and sepsis as compared to CDI patients without PTSD. A possible explanation for this finding may be a consequence of PTSD patients having an altered HPA axis [9]. Multiple prior studies have found that patients with PTSD have lower serum cortisol levels during stressful events as compared to people without PTSD [9]. While the pathophysiology explaining the lower cortisol levels in PTSD patients is not well understood, the limited literature on the subject posits that a low cortisol level is by itself a predisposing risk factor for the development of PTSD [9]. The inability to mount a normal stress response along with hypovolemia from sepsis in the setting of CDI may explain the increased inpatient mortality outcome seen in the PTSD subgroup. 

While PTSD has been associated with a low cortisol state, prior research has revealed that patients with PTSD have elevated levels of pro-inflammatory cytokines compared to healthy controls in the setting of a chronic hyperarousal state [15,16]. More specifically, pro-inflammatory cytokines interleukin-2, interleukin-6, and tumor necrosis factor-alpha have been observed to be elevated in PTSD patients [17]. Sepsis is an inflammatory state that is often triggered by infection [18]. A baseline inflammatory state in PTSD patients coupled with the inflammation from CDI may explain why this subgroup is at increased risk to develop sepsis. In addition, increased plasma concentrations of cytokines in the setting of sepsis is associated with a higher mortality state and may explain why the PTSD group experienced increased inpatient mortality [19].

In addition, it has been previously established that patients with PTSD have poor self-reported physical health, and increased rates of cardiovascular, respiratory, gastrointestinal, inflammatory, and autoimmune diseases [11]. These prior findings of increased comorbidities in patients with PTSD may initially seem to contradict the lower Charlson Comorbidity Index seen in Table 1. This is likely explained by the PTSD subgroup in this study being significantly younger than the average CDI patient without a history of PTSD. Most notably, the relationship between PTSD and increased rates of gastrointestinal diseases may contribute to the increased inpatient mortality and sepsis in the PTSD subgroup. PTSD has been found to impact the disease activity of gastrointestinal pathologies including IBD and IBS. It has been theorized that PTSD triggers gastrointestinal inflammation and dysregulates the brain-gut axis, which leads to increased IBD activity [20]. Similar to IBD, CDI represents a state of intestinal inflammation, so PTSD has the potential to exacerbate the intestinal inflammation induced by CDI [1].

There were several key limitations of the study. A significant limitation relates to the functionality of conducting database research with NIS. The NIS data relies on correct and accurate billing codes being entered by healthcare providers. Imprecise or inaccurate billing codes input by healthcare professionals can result in the over or underrepresentation of the PTSD subgroup of patients presenting with CDI in addition to outcomes assessed in this study. Also, the underdiagnosis of PTSD is a well-documented issue, and subsequently further decreases the number of patients who would fall into the PTSD subgroup within this study [21]. Another limitation of the study is the inability to characterize the severity of the disease, or the treatment modalities utilized given the limited ICD-9 codes for CDI and PTSD as well as the absence of ICD-9 codes for the therapeutics used. Despite the limitations, there were several important strengths of this study. First, while the ICD-9 code for CDI does not distinguish the symptomatic disease from asymptomatic colonization, prior studies that investigated the use of the ICD-9 code for CDI found that the ICD-9 code for CDI is close to a true approximation of infection [22,23]. A second important strength of the study is the ability to evaluate patient outcomes and demographics on a nationwide scale. A final noteworthy strength of this study is the utilization of a multivariate logistic regression analysis that adjusts for numerous potential confounding variables. 

## Conclusions

In summary, hospitalized patients with a history of PTSD and a diagnosis of CDI have an increased risk of sepsis and inpatient mortality. Given the elevated risk associated with PTSD in patients with CDI, it may be necessary to adjust the management of these patients, for example keeping the patients hospitalized and delaying discharge until the patients have demonstrated a substantial response to antibiotics for CDI. In addition, having a low threshold to escalate the level of care of CDI patients with comorbid PTSD may be necessary if any early signs of sepsis are present or if the patients have other medical comorbidities that increase their risk of inpatient mortality. Further research is required to investigate whether a relationship exists between the severity of the PTSD, or how well-controlled the PTSD is, and the frequency of the outcomes in this study. If there is a relationship, coordinating with psychiatrists to increase PTSD screening and optimizing therapeutic regimens may have a role to decrease the potential to decrease both sepsis and inpatient mortality due to CDI.
